# Matrix Metalloproteinase-2 Isoforms Differ within the Aortic Wall of Ascending Aortic Aneurysms Associated with Bicuspid Aortic Valve

**DOI:** 10.1155/2020/1306425

**Published:** 2020-09-23

**Authors:** Ramona Schmitt, Anke Tscheuschler, Philipp Laschinski, Philipp Discher, Jana Fuchs, Fabian A. Kari

**Affiliations:** ^1^Department of Cardiovascular Surgery, University Heart Center Freiburg-Bad Krozingen, University of Freiburg, Freiburg, Germany; ^2^Faculty of Medicine, University of Freiburg, Freiburg, Germany

## Abstract

The pathogenesis of ascending thoracic aortic aneurysm (aTAA) is thought to differ between patients with bicuspid aortic valve (BAV) and tricuspid aortic valve (TAV), and one of the causes is different hemodynamics. Influenced by hemodynamics, the tissue levels of proteins associated with aTAA might differ between aTAAs with BAV and TAV and between different localities within the aortic wall. We therefore analyzed aTAA tissue levels of MMP-2 (matrix metalloproteinase-2) isoforms (Pro-MMP-2, active MMP-2, and total MMP-2) and tissue levels of MMP-14, TIMP-2 (tissue inhibitor of metalloproteinase-2), MMP-9, and TIMP-1 in 19 patients with BAV and 23 patients with TAV via gelatin zymography and enzyme-linked immunosorbent assay (ELISA), respectively. TAV and BAV groups' protein levels did not differ significantly. Whereas the TAV group exhibited no significant differences in protein levels between the aneurysm's anterior and posterior parts, the BAV group revealed significantly higher levels of Pro-MMP-2, total MMP-2, and TIMP-2 in the aneurysm's posterior parts (mean Pro-MMP-2 200.52 arbitrary units (AU) versus 161.12 AU, *p*=0.007; mean total MMP-2 235.22 AU versus 193.68 AU, *p*=0.002; mean TIMP-2 26.90 ng/ml versus 25.36 ng/ml, *p*=0.009), whereas the other proteins did not differ significantly within the aortic wall. Thus, MMPs are distributed more heterogeneously within the aortic wall of aTAAs associated with BAV than in those associated with TAV, which is a new aspect for understanding the underlying pathogenesis. This heterogeneous protein level distribution might be attributable to differences in the underlying pathogenesis, especially hemodynamics. This result is important for further studies as it will be essential to specify the location of samples to ensure data comparability regarding the main goals of understanding the pathogenesis of aTAA, optimizing treatments, and establishing a screening method for its potentially deadly complications.

## 1. Introduction

The aortic valve's morphology (BAV or TAV) is believed to be an influencing factor in the development of ascending thoracic aortic aneurysms (aTAAs).

The BAV is the most frequent congenital valvular defect with an incidence of approximately 1% and is associated with aTAA [1]. The pathogenesis of aTAAs associated with BAV seems to differ from that of aTAAs associated with TAV; it is determined by genetic and hemodynamic factors [1–3]. Furthermore, the risk of dissection or aortic rupture of an aTAA with BAV is about eight times higher than the risk of an aTAA with TAV [4, 5].

However, the underlying pathogenesis of an aTAA associated with BAV and with TAV is still not fully understood, although the influencing factors have been discussed such as matrix metalloproteinases (MMPs), especially MMP-2 and MMP-9, due to their active form's ability to degrade elastin and collagen [6].

We therefore conducted this study to address whether differences in the pathogenesis of aTAAs associated with BAV and TAV result in differences in MMP protein levels and their regulating proteins, the tissue inhibitors of metalloproteinases (TIMPs).

Considering influencing factors, genetics and—directly linked to valve morphology—especially hemodynamics in aTAA with BAV, we can assume that protein levels would differ from location to location in the aortic wall. It remains unclear whether the MMP-2 isoforms (Pro-MMP-2, active MMP-2, and total MMP-2), as well as MMP-9 and MMP-2 activating and regulation proteins TIMP-2 and MMP-14, differ according to their locations within the aortic wall of aTAA and whether such a potential difference depends on the aortic valve's morphology.

Our aim was thus to analyze the MMP-2 isoforms, MMP-2 influencing MMP-14 and TIMP-2, and aTAA influencing MMP-9 and TIMP-1 by comparing patients with BAV to TAV and further to evaluate any potential difference in the local distribution of protein levels within the aortic wall of aneurysms (anterior versus posterior part) associated with BAV and TAV, respectively.

## 2. Materials and Methods

### 2.1. Study Design and Patient Characteristics

All recruited patients were participants in this clinical study “Biomarkers of Shear Stress and Wall Tension in Thoracic Aortic Aneurysms” (German Clinical Trial Register-ID: DRKS00004866, https://www.drks.de). Our study group consisted of 42 patients with an aneurysm in the aortic root and/or ascending aorta. The study included patients aged 18 to 85 years with bicuspid or tricuspid aortic valves, and no selection was made regarding the Sievers classification [7] or aortic valve function (normal valve function, stenosis, insufficiency, and combined valve dysfunction). Exclusion criteria were any active malignancy, children or age above 85 years, any earlier stent graft intervention or prosthetic replacement on the aorta, and aortic dissection of any kind. Our study group was further subdivided into two groups: patients with TAV (*n* = 23) and those with BAV (*n* = 19).

Our patients' characteristics are summarized in Table 1. This study was approved by the Ethics Committee of the University of Freiburg, and all patients provided informed written consent.

### 2.2. Sample Preparation and Protein Extraction

**Figure 1:**
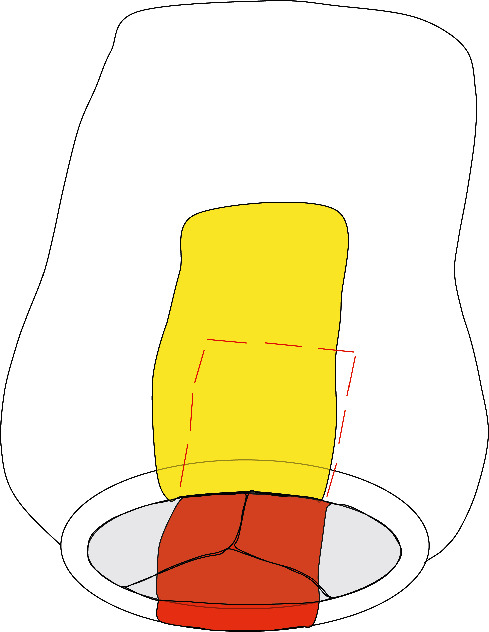
Locations of tissue extracted from the ascending aorta: view from the bottom of the aortic valve, anterior tissue displayed in yellow and posterior tissue displayed in red and dotted line.

Sample preparation and experiments were carried out and validated as described previously by our group [8, 9]. Aortic tissue from the aneurysm's anterior and posterior parts (Figure 1) was deep frozen in liquid nitrogen immediately after resection and stored at −80°C.

To extract proteins, the entire aortic-tissue sample was pulverized in liquid nitrogen and then supplied with ice-cold lysis buffer (50 mM Tris, 150 mM NaCl, 1% Triton X-100, pH 7.5) containing protease inhibitor P8340 (Sigma). Samples were incubated on ice for one hour and centrifuged for 15 minutes at 13.000 rpm and 4°C. Supernatant was filtered through spin-x-centrifuge filters (0.22 *μ*m cellulose acetate, Costar) by centrifugation and pellets were resuspended in lysis buffer, followed by incubation for 30 minutes, centrifugation, and filtration. The protein extracts were aliquoted and stored at −20°C. Protein extracts for the MMP-14-ELISA were drawn using the 1X Cell Extraction Buffer PTR (supplied by the ELISA Kit), supplied with protease inhibitor P8340. Extraction steps were the same as those mentioned above.

Total protein concentrations were determined via bicinchoninic acid assay (BCA) following the manufacturer's instructions (Thermo Scientific Pierce BCA Protein Assay).

### 2.3. Gelatin Zymography

Protein extracts were diluted with zymography buffer (25 mM Tris, 150 mM NaCl, 10 mM CaCl_2_, 0.2% Brij-35, pH 7.5) containing protease inhibitor (P8340, Sigma) and a total protein amount of 15 *μ*g was loaded onto 8% SDS gels containing 0.2% gelatin (gelatin from porcine skin G1890, Sigma). Electrophoresis was performed at 20 mA per gel for 2.5 hours. Gels were washed twice for 30 minutes with 2.5% Triton X-100 at room temperature with agitation, followed by incubation in zymography buffer at 37.2°C for 19 hours during which gelatin digestion occurred. Afterward, the gels were stained with 50 mL 0.2% Coomassie Brilliant Blue R-250 (Serva) following the manufacturer's instructions.

Pro-MMP-2 and active MMP-2 in the samples were identified via a human full-length MMP-2 protein (ab168864, Abcam) (as described previously by our group [8, 9]) and were semiquantitatively determined by analyzing pixel density with software Image J (version 1.47, Wayne Rasband, National Institutes of Health, USA). Each sample was normalized to 0.33 ng human full-length MMP-2. All samples were measured three times independently, and those findings were averaged. MMP-2's total protein level was calculated by summarizing Pro-MMP-2 and active MMP-2 of the corresponding sample.

### 2.4. Enzyme-Linked Immunosorbent Assay (ELISA)

MMP-14, TIMP-2, MMP-9, and TIMP-1 tissue levels were quantified using sandwich enzyme-linked immunosorbent assay (ELISA) kits. A standard curve was run in each assay; all samples and standards were measured in duplicate and findings were averaged. The TIMP-2, MMP-9, and TIMP-1 assay procedures were done according to the manufacturer's instructions (DTM200, DMP900, and DTM100, R&D Systems). The MMP-14 assay procedure was done according to the manufacturer's instructions with the samples in the antibody cocktail's incubation time increasing to two hours (ab197747, Abcam).

### 2.5. Statistical Analysis

Statistical analysis was performed using SigmaPlot version 13.0 (Systat Software GmbH, Erkrath, Germany). Data was tested for normal distribution by the Shapiro-Wilk test. We also calculated means and standard deviations. To compare the BAV and TAV groups' total protein levels, we averaged data from each aneurysm's anterior and posterior parts. The two groups were compared applying the *t*-test or Mann–Whitney rank sum test. Categorical variables between the BAV and TAV groups were compared by Fisher's exact test or *χ*^2^ test. A *p*-value < 0.05 was considered significant.

## 3. Results

Our analysis of patient characteristics (Table 1) showed that the BAV group's mean age was 57.38 years (standard deviation (SD) 11.65), consisting of 3 (15.8%) female and 16 (84.2%) male patients. Their mean ascending-aorta diameter was 51.42 mm (SD 4.65). In this group, 1 patient revealed no aortic valve pathologies, 3 patients had aortic stenosis, 4 had aortic insufficiency, and 11 had combined valve dysfunction. The Sievers BAV classification ([7]) showed that 15 patients presented a type 1 L-R and 2 patients presented a type 1 N-R, but no reliable classification was possible due to severely impaired valves in 2 patients.

The TAV group's mean age was 64.00 years (SD 14.50); the group consisted of 7 women (30.4%) and 16 men (69.6%). Mean ascending-aorta diameter measured 55.35 mm (SD 8.41). In this group, 3 patients exhibited no aortic valve pathologies, 2 had had aortic stenosis, 15 had aortic insufficiency, and 3 had combined valve dysfunction.

The two groups' mean age and mean ascending-aorta diameter did not differ significantly (*p*=0.116 and *p*=0.141, resp.).

Patients with BAV presented combined valve dysfunction significantly more often (*p*=0.003), whereas TAV patients suffered from aortic valve insufficiency significantly more often (*p*=0.006).

Regarding the patients' comorbidities, comparing the two groups revealed no significant difference in the prevalence of hypertension, hyperlipidemia, diabetes mellitus, or connective tissue disorders (*p* > 0.300 each).

## 4. Results of Zymography and ELISA

Our zymographic and ELISA results are summarized in Table 2.

A meaningful analysis of protein levels comparing aortic insufficiency, stenosis, normal valve function, and combined valve dysfunction between and within the two groups was unfeasible because our subgroups were so small (resulting in unsatisfactory test power).

### 4.1. No Differences in Protein Levels between Patients with BAV and Those with TAV

**Figure 2:**
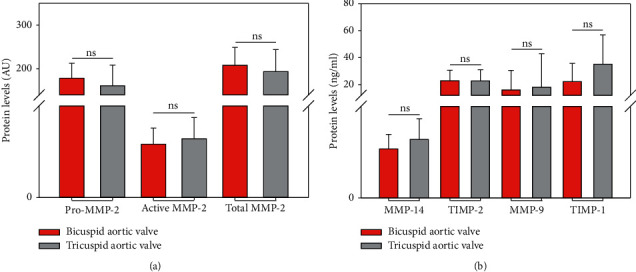
Comparison of protein levels between patients with BAV and patients with TAV (MMP-2 isoforms given in AU and ELISA results given in ng/ml; ns: nonsignificant).

Comparative analysis of our results revealed no significant difference between BAV and TAV patients in total protein levels (anterior and posterior levels averaged) (Pro-MMP-2, *p*=0.200; active MMP-2, *p*=0.349; total MMP-2, *p*=0.328; MMP-14, *p*=0.096; TIMP-2, *p*=0.981; MMP-9, *p*=0.649; TIMP-1, *p*=0.077) (Figure 2).

These results are strengthened by comparing the BAV and TAV groups' anterior parts (Pro-MMP-2, *p*=0.980, active MMP-2, *p*=0.145, total MMP-2, *p*=0.797, MMP-14, *p*=0.411, TIMP-2, *p*=0.615, MMP-9, *p*=0.742, and TIMP-1, *p*=0.061) and posterior parts (Pro-MMP-2, *p*=0.058, active MMP-2, 0.879, total MMP-2, *p*=0.095, MMP-14, *p*=0.622, TIMP-2, *p*=0.671, MMP-9, *p*=0.940, and TIMP-1, *p*=0.075), where the two groups revealed no significant differences in various locations.

### 4.2. Different MMP-2 Protein Levels in the Anterior and Posterior Parts of the Ascending Aortic Wall in Patients with BAV

**Figure 3:**
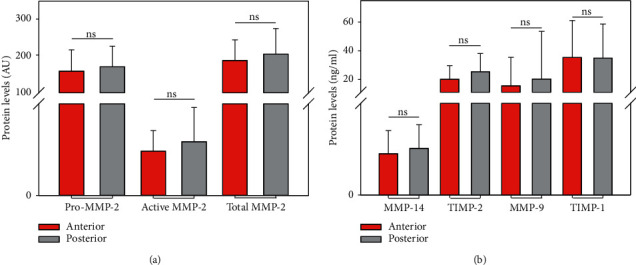
Comparison of protein levels between the anterior and the posterior parts of the aneurysms associated with TAV (MMP-2 isoforms given in AU and ELISA results given in ng/ml; ns: nonsignificant).

Analysis of our TAV group's data showed no significant difference in the protein levels between the aneurysm's anterior and posterior parts (Pro-MMP-2, *p*=0.498, active MMP-2, *p*=0.416, total MMP-2, *p*=0.373, MMP-14, *p*=0.244, TIMP-2, *p*=0.121, MMP-9, *p*=0.227, and TIMP-1, *p*=0.860) (Figure 3).

**Figure 4:**
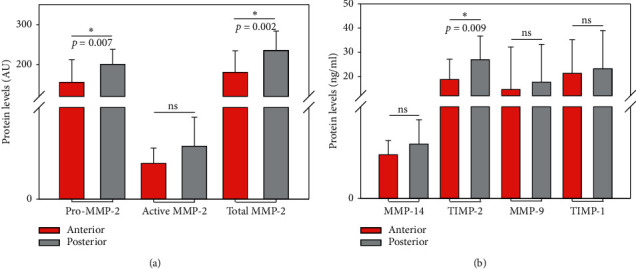
Comparison of protein levels between the anterior and posterior parts of the aneurysms associated with BAV (MMP-2 isoforms given in AU and ELISA results given in ng/ml; ns: nonsignificant).

In contrast, our analysis of the BAV group's zymographic data revealed significantly higher Pro-MMP-2 and total MMP-2 levels in the aneurysm's posterior part compared to its anterior part (*p*=0.007 versus *p*=0.002). However, the two parts' active MMP-2 levels did not differ significantly (*p*=0.096). The BAV group's ELISA results showed no significant differences in MMP-14, MMP-9, and TIMP-1 levels between anterior and posterior parts (MMP-14, *p*=0.064, MMP-9, *p*=0.414, and TIMP-1, *p*=0.748). TIMP-2 levels were significantly higher in the posterior part of the aneurysms associated with BAV (*p*=0.009) (Figure 4).

## 5. Discussion

Our study aimed to analyze potential differences in MMP levels between patients with BAV and TAV aTAA by emphasizing any potential differences between the aneurysm's anterior and posterior parts.

Our patients with BAV exhibited no significant differences in age or ascending-aorta diameter compared to the patients with TAV, a finding that contradicts other studies' results [10]. Evidence is accumulating that patients with BAV develop aTAAs when younger, that their aneurysms progress faster, and that they carry a higher dissection risk [4, 11]. Our results are attributable to the silent character of aTAAs, as they are usually asymptomatic and detected coincidentally through clinical diagnostics in conjunction with other symptoms [12]. Furthermore, our findings highlight the need for a noninvasive method to screen for aTAA and for dissection-risk evaluations, especially in patients with BAV.

Patients with BAV presented combined valve disease significantly more often, while those with TAV revealed aortic valve insufficiency significantly more often. The most frequent complication of BAV is aortic stenosis [1] while a dilated ascending aorta is known to trigger secondary aortic valve insufficiency [13]. Thus, a potential explanation for the aforementioned differences in valve pathologies between patients with BAV and TAV is that BAV patients develop an aortic stenosis first, which leads to aTAA development, and that in turn leads to a secondarily insufficient aortic valve and then combined valve disease. In contrast, our data from our TAV patients imply a different pathogenesis leading to aTAA and an insufficient aortic valve.

Our findings from the MMPs analyzed in this study might explain some of the aforementioned differences between the aTAAs in patients with BAV and TAV.

Since the BAV and TAV groups exhibited no significant differences in either total protein levels or local protein levels (anterior and posterior), we conclude that the aortic valve's number of leaflets does not directly influence total protein expression. Furthermore, neither the total amount of MMP-2 activation nor influencing proteins MMP-14 and TIMP-2 in this process [14] seem to differ between BAV and TAV patients.

This result also stands in contrast to studies that reported significantly increased total and active MMP-2 and MMP-9 and decreased MMP-14 in patients with BAV compared to those with TAV [15–18]. A limiting factor in those studies is that they failed to state which part of the aortic wall they examined, and in most of the cases, total MMP-2 was measured rather than the isoforms.

However, our results find support in an ex vivo study showing no significant differences between MMP-2 isoforms and MMP-9 in the concave part of fluid-structure interaction models of ascending aneurysms exposed to TAV and BAV flows or wall shear stress [19]. Moreover, another investigation reported no differences in the gene expression of MMP-2, MMP-9, TIMP-1, and TIMP-2 between BAV and TAV aTAAs [20].

These divergent study findings may be due to differences in the pathogenesis of aneurysms with BAV and those with TAV, which are incompletely understood [3]. It is thus essential to understand the precise mechanisms of aTAA development, especially the influence of aortic valve hemodynamics and morphology; we also need to reevaluate whether differences between studies can be attributed to underlying pathogenetic mechanisms.

Despite the similar total and local protein levels we observed between BAV and TAV patients, the subgroup of patients with BAV displayed significant local differences in the protein levels of Pro-MMP-2, total MMP-2, and TIMP-2, with higher protein levels in the posterior part, whereas TAV patients did not. Hence, while the two groups' total protein expressions do not seem to differ, their local protein expressions do in how they are distributed and regulated, with more heterogeneously distributed MMPs in aTAAs associated with BAV.

These results lead us to pose the following key questions:

1. What causes significant differences in Pro-MMP-2, total MMP-2, and TIMP-2 within the aortic wall without influencing the other proteins, and why are the other proteins, especially active MMP-2, unaffected?
2. Why do these differences appear in aneurysms associated with BAV only?
3. Why are protein levels significantly increased in the aneurysm's posterior part?

A potential explanation for protein levels' differences within the aortic wall is altered hemodynamics within these aneurysms. Possible causes for altered hemodynamics are, for example, previous stent-implantation (and consecutive alterations in electrical parameters of erythrocyte membranes) [21] or differences in wall shear stress.

Various working groups have demonstrated differences in wall shear stress between patients with BAV and TAV [2, 22, 23]. Increased wall shear stress causes enhanced degradation of the medial layer and increased MMP-2 and MMP-9, as well as their active forms [24–27]. There is also evidence that the mRNA expression of TIMP-1, TIMP-2, and MMP-14 is unaffected by mechanical stretch, a finding in line with our TIMP-1 and MMP-14 analysis results [26].

Thus, differences in wall shear stress might explain our results, indicating that the differences in aTAAs with BAV are strong enough to trigger differences in some protein levels, whereas other protein levels are not (yet) influenced. In contrast, the differences in aTAAs with TAV do not seem strong enough to result in differences in measured protein levels.

Enhanced wall shear stress in BAV and TAV aTAA has been especially apparent in the aortic wall's anterolateral region, with greater stress in BAV [23, 28]. Considering the aforementioned literature, this should result in enhanced activation of MMP-2 and MMP-9 in those aneurysm regions.

Our BAV Pro-MMP-2, total MMP-2, and TIMP-2 findings comparing the aneurysm's anterior and posterior parts concur with this hypothesis. As MMP-9 revealed no significant difference between anterior and posterior parts, it seems possible that influencing factors other than hemodynamics may be playing an important role in regulating this protein. Concerning the *p*-value of active MMP-2 and MMP-14 comparing anterior and posterior parts within BAV aTAA (*p*=0.096; *p*=0.064), we cannot entirely rule out a difference within the aortic wall in a larger patient cohort.

However, our analysis of these proteins in BAV also enables the hypothesis that MMP-2 activation differs within the aortic wall of patients with BAV, as there were no significant differences in active MMP-2 between the anterior and posterior parts, but there were significant differences in Pro-MMP-2 and TIMP-2-two proteins influencing MMP-2 activation [14]. This indicates that a higher percentage of Pro-MMP-2 is activated in the aneurysm's anterior part, suggesting increased proteolysis there. Increased proteolysis in the aneurysm's anterior part harmonizes with the hypothesis of increased wall shear stress and increased MMP-2 activation, although this does not seem significantly evident in active MMP-2 levels.

Our TAV results do not depict the local maximum wall shear stress in the aneurysm's anterolateral region, a finding attributable to the fact that wall shear stress in TAV aTAAs is believed to be more symmetrical [29]; thus, MMPs' induction does not appear to differ significantly, and their distribution seems to be more homogeneous within the aortic wall.

One study already investigated MMP-2 and MMP-9 within the concave and convex parts of aTAA, demonstrating that total MMP-2 was significantly higher in the concave part, while MMP-9 was significantly higher in the convex part [30].

Considered together with our findings revealing differences between the anterior and posterior parts in patients with BAV, this implies that protein levels within the aTAA aortic wall possess a complex distribution pattern, especially in patients with BAV.

More investigations are therefore needed addressing these questions: do protein levels and their expression within the aortic wall reveal a certain pattern and does that pattern correlate with hemodynamics and the risk of aneurysm rupture? Is this pattern somehow detectable in peripheral blood, which could then serve as a biomarker to screen for aTAA?

Significant local differences are a limiting factor when seeking biomarkers for aTAA in peripheral blood. We simply do not yet know whether one or more markers can depict local protein levels if there are already significant differences within the aortic wall before those proteins are washed out into peripheral blood.

As a study limitation, we were unable to compare our findings with a control group possessing nonaneurysmatic ascending aortic tissue. Our clinical study included control patients undergoing coronary artery bypass surgery [9], but all those tissue samples were taken from the ascending aorta's anterior part (central anastomosis). We therefore could not compare anterior and posterior protein levels in patients with BAV and TAV with those in control patients.

Another remaining question is whether, and if so, to what extent, aortic valve function (stenosis, insufficiency, combined dysfunction, and normal function) and the BAV's cusp fusion pattern influence protein levels otherwise. There is ample evidence of differences in MMPs and wall shear stress between various BAV subtypes, as well as differences in the aortic media comparing the BAV's aortic valve function [31–34]. As our groups subdivided according to aortic valve function were too small, we could not analyze our data to clarify this point.

A comparison of anterior and posterior protein levels with control aortic tissue as well as investigating the influence of aortic valve function and cusp fusion patterns should be considered starting points for future studies seeking to clarify the pathogenesis of ascending aortic aneurysms in patients with BAV and TAV.

In summary, although there are no significant differences between the total protein levels of MMP-2 isoforms, MMP-9, MMP-14, TIMP-1, and TIMP-2 between aTAAs associated with BAV and TAV, the extent to which these proteins are distributed within the aortic wall does differ, especially in patients with BAV.

## 6. Conclusions

Our study found that total MMP levels do not differ between aTAAs in patients with BAV and TAV. However, analyses of these protein levels in the anterior and posterior parts of these aTAAs suggest a complex, heterogeneous distribution of MMPs within the aortic wall of aTAAs associated with BAV, while the distribution of MMPs seems to be more homogeneous in aTAAs associated with TAV. These differences might be caused by differences in the pathogenesis of aTAAs associated with BAV and TAV, especially hemodynamic differences known to induce MMP expression and activation. It is essential that future studies specify the location of resected samples to ensure the comparability of data on the main goal of understanding the pathogenesis of aTAA, optimizing treatments, and establishing a screening method for its potentially deadly complications.

## Figures and Tables

**Table 1:** Patients' characteristics.

Characteristics	Factor
*Bicuspid aortic valve*	
Number of patients (% of study group)	*N* = 19 (45.2)
Age (years) (mean (SD))	57.38 (11.65)
Gender (%)	
Female	*N* = 3 (15.8)
Male	*N* = 16 (84.2)
Ascending-aorta diameter (mm) (mean (SD))	51.42 (4.65)
Aortic valve function (%)	
Normal/physiologic	*N* = 1 (5.2)
Stenosis	*N* = 3 (15.8)
Insufficiency	*N* = 4 (21.1)
Combined valve dysfunction	*N* = 11 (57.9)
Sievers classification (%) [7]	
Type 0	*N* = 0 (0)
Type 1 L-R	*N* = 15 (79.0)
Type 1 L-N	*N* = 0 (0)
Type 1 R-N	*N* = 2 (10.5)
Type 2	*N* = 0 (0)
Not reliably classifiable	*N* = 2 (10.5)
Comorbidities	
Hypertension (%)	*N* = 10 (52.6)
Hyperlipidemia (%)	*N* = 9 (47.4)
Diabetes mellitus (%)	*N* = 0 (0)
Connective tissue disorders (%)	*N* = 0 (0)

*Tricuspid aortic valve*	
Number of patients (% of study group)	*N* = 23 (54.8)
Age (years) (mean (SD))	64.00 (14.50)
Gender (%)	
Female	*N* = 7 (30.4)
Male	*N* = 16 (69.6)
Ascending-aorta diameter (mm) (mean (SD))	55.35 (8.41)
Aortic valve status (%)	
Normal valve function	*N* = 3 (13.0)
Stenosis	*N* = 2 (8.7)
Insufficiency	*N* = 15 (65.3)
Combined valve dysfunction	*N* = 3 (13.0)
Comorbidities	
Hypertension (%)	*N* = 15 (65.2)
Hyperlipidemia (%)	*N* = 10 (43.5)
Diabetes mellitus (%)	*N* = 3 (13.0)
Connective tissue disorders (%)	*N* = 2 (8.7)^*∗*^

SD: standard deviation; ^*∗*^*n* = 1 Marfan syndrome and *n* = 1 alpha-actin-2 mutation.

**Table 2:** Results of zymography and ELISA (MMP-2 isoforms given in AU and MMP-14, TIMP-2, MMP-9, and TIMP-1 given in ng/ml).

Protein	Mean protein level (SD) BAV	TAV
Pro-MMP-2		
Anterior	155.71 (56.63)	155.25 (59.31)
Posterior	200.52 (37.54)	167.00 (57.39)
Averaged	178.11 (34.73)	161.12 (47.19)
Active MMP-2		
Anterior	23.42 (10.05)	28.98 (13.47)
Posterior	34.70 (18.89)	35.25 (22.37)
Averaged	29.06 (8.81)	32.12 (11.54)
Total MMP-2		
Anterior	180.66 (53.64)	185.11 (56.00)
Posterior	235.22 (48.69)	202.25 (71.42)
Averaged	207.94 (41.05)	193.68 (50.39)
MMP-14		
Anterior	2.86 (0.93)	3.61 (2.02)
Posterior	3.56 (1.59)	4.06 (2.07)
Averaged	3.21 (0.95)	3.84 (1.35)
TIMP-2		
Anterior	18.72 (8.40)	20.13 (9.45)
Posterior	26.90 (9.84)	25.36 (12.78)
Averaged	22.81 (7.81)	22.75 (8.31)
MMP-9		
Anterior	14.56 (17.63)	15.56 (19.93)
Posterior	17.60 (15.68)	20.32 (33.24)
Averaged	16.08 (14.45)	17.94 (24.98)
TIMP-1		
Anterior	21.23 (14.01)	35.30 (25.75)
Posterior	23.13 (15.86)	34.82 (23.82)
Averaged	22.18 (13.52)	35.06 (21.81)

BAV: bicuspid aortic valve; TAV: tricuspid aortic valve; AU: arbitrary units; SD: standard deviation.

## Data Availability

The datasets used and/or analyzed during the current study are available from the corresponding author upon request.
